# Aqueous Extract of *Buddleja globosa* Modulates Oxidative Stress-Induced Senescence-Associated Responses in THP-1-Derived Macrophages

**DOI:** 10.3390/ph19091447

**Published:** 2026-09-12

**Authors:** Roberto Bravo-Sagua, Jakelin Gallardo, Daniela Bergez, Alina Concepción-Alvarez, Raquel Bridi, Adriano Costa de Camargo, Jocelyn Fuentes, Hernán Speisky, Pedro Cisternas, Nicolás Tobar, Sergio Wehinger, Felipe Avila, Isidora Aguirre-Becerra, Paulina Ormazabal

**Affiliations:** 1 Laboratory of Obesity and Metabolism (OMEGA), Instituto de Nutrición y Tecnología de los Alimentos (INTA), Universidad de Chile, El Líbano 5524, Macul, Santiago 7830490, Chile; rbravosagua@inta.uchile.cl (R.B.-S.); jakelin.gallardo@usach.cl (J.G.); daniela.bergez@usach.cl (D.B.); 2 Advanced Center for Chronic Diseases (ACCDiS), Universidad de Chile, Santiago 8380492, Chile; 3 Interuniversity Center for Healthy Aging RED21993, Santiago 8320216, Chile; snunez@utalca.cl (S.W.); favilac@utalca.cl (F.A.); 4 Instituto de Nutrición y Tecnología de Los Alimentos (INTA), Universidad de Chile, El Líbano 5524, Macul, Santiago 7830490, Chile; a24ca90@gmail.com (A.C.-A.); adrianodecamargo@inta.uchile.cl (A.C.d.C.); 5 Departamento de Química Farmacológica y Toxicológica, Facultad de Ciencias Químicas y Farmacéuticas, Universidad de Chile, Dr. Carlos Lorca Tobar 964, Independencia, Santiago 8380492, Chile; raquelbridi@ciq.uchile.cl; 6 Instituto de Ciencias Aplicadas, Universidad Autónoma de Chile, Providencia, Santiago 7500910, Chile; 7 Laboratory of Antioxidants, Nutrition and Food Technology Institute, University of Chile, El Líbano 5524, Macul, Santiago 7830490, Chile; jfuentes@inta.uchile.cl (J.F.); hspeisky@inta.uchile.cl (H.S.); 8 Núcleo de Investigación en Nutrición y Ciencias Alimentarias (NINCAL), Facultad de Salud y Ciencias Sociales, Universidad de las Américas, Av. Manuel Montt 948, Providencia, Santiago 7500658, Chile; pcisternasf@udla.cl; 9 Cellular Biology Laboratory, Instituto de Nutrición y Tecnología de los Alimentos (INTA), Universidad de Chile, El Líbano 5524, Macul, Santiago 7830490, Chile; ntobar@inta.uchile.cl; 10 Thrombosis and Healthy Aging Research Center, Medical Technology School, Faculty of Health Sciences, Interuniversity Center for Healthy Aging, Universidad de Talca, Talca 3480094, Chile; 11 Departamento de Ciencias de la Nutrición y los Alimentos, Escuela de Nutrición y Dietética, Facultad de Ciencias de la Salud, Universidad de Talca, Talca 3460000, Chile; 12 Escuela de Medicina, Facultad de Medicina y Ciencia, Universidad San Sebastián, Lota 2465, Providencia, Santiago 7500000, Chile; iaguirreb@correo.uss.cl; 13 Escuela de Obstetricia, Facultad de Ciencias para el Cuidado de la Salud, Universidad San Sebastián, Lota 2465, Providencia, Santiago 7510157, Chile

**Keywords:** *Buddleja globosa*, oxidative stress, cellular senescence, THP-1-derived macrophages, NF-κB, botanical extract

## Abstract

**Background/Objectives**: Cellular senescence and oxidative stress contribute to macrophage dysfunction and chronic inflammation during aging. *Buddleja globosa* (matico) is traditionally consumed as an infusion and has reported antioxidant and anti-inflammatory properties. This study evaluated whether an aqueous extract of *B. globosa* (BgAE) modulates senescence-associated responses in THP-1-derived macrophages exposed to hydrogen peroxide. **Methods:** The phenolic profile of BgAE was analyzed by HPLC-DAD and its antioxidant capacity by the oxygen radical absorbance capacity assay. Cell viability was assessed after exposure to 0.01–50 µg/mL BgAE. THP-1-derived macrophages were pretreated with BgAE for 48 h, exposed to 50 µM H_2_O_2_ for 1 h, and allowed to recover for 5 days. Senescence-associated β-galactosidase, inflammatory gene expression, and p16, p21, phosphorylated NF-κB, and total NF-κB protein levels were evaluated. **Results:** BgAE showed antioxidant capacity and did not significantly reduce cell viability under the conditions tested. H_2_O_2_ increased senescence-associated β-galactosidase positivity, *TNFA* expression, p16 and p21 abundance, and NF-κB phosphorylation. BgAE reduced H_2_O_2_-induced β-galactosidase positivity at 1 and 50 µg/mL and modulated *TNFA*, p16, p21, and NF-κB responses, with effects varying across the concentrations evaluated. The 0.01 and 1 µg/mL conditions generally showed greater attenuation of H_2_O_2_-induced changes, whereas 50 µg/mL BgAE alone increased senescence-associated signaling. **Conclusions:** BgAE modulates selected senescence-associated and inflammatory responses in oxidatively stressed THP-1-derived macrophages. These findings provide initial in vitro evidence supporting further investigation and improved standardization of *B. globosa* aqueous preparations as modulators of oxidative stress-associated cellular responses.

## 1. Introduction

Aging is a complex biological process characterized by the progressive loss of cellular and tissue homeostasis [1]. A prominent feature of aging is the development of chronic, low-grade inflammation, commonly referred to as inflammaging, which contributes to functional decline and the development of age-related disorders [2,3]. Macrophages are important regulators of tissue homeostasis and inflammatory responses; however, aging alters their phenotype and function, favoring persistent inflammatory signaling, impaired stress adaptation, and defective resolution of inflammation [2,4]. Accumulation of dysfunctional or senescent macrophages may therefore reinforce local and systemic inflammation and contribute to the progressive deterioration of tissue function during aging [4,5,6].

Cellular senescence is a stress-associated cellular state characterized by a coordinated but heterogeneous set of phenotypic and molecular alterations rather than by a single universal marker [7,8]. Commonly evaluated features include increased senescence-associated β-galactosidase (SA-β-Gal) activity and the upregulation of the cyclin-dependent kinase inhibitors p16INK4a and p21Cip1/Waf1, which are associated with senescence-related cell-cycle control [4,7,9]. Senescent cells may also develop a senescence-associated secretory phenotype (SASP), comprising proinflammatory cytokines, chemokines, growth factors, and matrix-remodeling mediators [4,10]. Importantly, the expression of these features varies according to cell type, differentiation state, senescence-inducing stimulus, and time after exposure; consequently, cellular senescence should be evaluated using multiple complementary markers [4,11].

Oxidative stress is an important inducer of stress-associated cellular senescence [12]. Persistent accumulation of reactive oxygen species can promote macromolecular damage and activate signaling pathways involved in cell-cycle regulation and inflammatory responses, including p16, p21, and NF-κB [13,14]. Consequently, antioxidant compounds have been investigated as potential modulators of senescence-associated phenotypes [15]. Plant-derived polyphenols are particularly relevant because, in addition to their radical-scavenging properties, they can influence redox-sensitive and proinflammatory signaling pathways, including NF-κB [14,16]. However, the biological effects of polyphenol-containing extracts may depend on their chemical composition, concentration, cellular context, and experimental conditions.

*Buddleja globosa*, commonly known as matico, is a South American medicinal plant traditionally used in Chile, particularly in the form of leaf infusions and topical preparations for the management of wounds and inflammatory conditions [17,18]. Phytochemical studies of *B. globosa* have described phenylethanoid glycosides, flavonoids, terpenoids, and other phenolic constituents, while experimental evidence supports antioxidant and anti-inflammatory activities of different preparations obtained from this plant [17,19,20]. Because the chemical composition and biological activity of botanical extracts may vary according to the plant material and extraction procedure [17,20,21], aqueous preparations are particularly relevant for investigating a formulation that resembles the traditional use of matico. However, the experimental preparation used here was not intended to reproduce a standardized ethnomedicinal formulation. Whether an aqueous extract of *B. globosa* modulates oxidative stress-induced senescence-associated responses in macrophages remains unknown.

Based on this rationale, the present study investigated whether an aqueous extract of *B. globosa* modulates oxidative stress-induced senescence-associated responses in THP-1-derived macrophages. Cells were pretreated with different concentrations of the extract and subsequently exposed to H_2_O_2_. The effects of the extract were evaluated by assessing cell viability, SA-β-Gal positivity, the expression of inflammatory genes, and the abundance of p16, p21, and phosphorylated NF-κB. We hypothesized that BgAE would attenuate selected senescence-associated and inflammatory responses induced by oxidative stress, with effects potentially varying according to extract concentration.

## 2. Results

### 2.1. Phenolic Profile and Antioxidant Capacity of the Buddleja globosa Aqueous Extract

First, we assessed the phenolic composition and antioxidant capacity of the 5% (*w*/*v*) *B. globosa* aqueous extract (BgAE). Among the compounds quantified by HPLC-DAD, rutin trihydrate, quercetin, and caffeic acid were the most abundant (Table 1). Quantitative HPLC-DAD results are expressed as mg per 100 g of dried botanical material used to prepare the 5% (*w*/*v*) aqueous extract. The sample analyzed by HPLC-DAD corresponded to the original liquid aqueous preparation and was injected without prior lyophilization. Representative HPLC-DAD chromatograms illustrating the chromatographic separation and retention times of the quantified compounds are provided in Supplementary Figure S1, together with the available analytical information supporting compound identification and quantification in Supplementary Table S1. The same 5% (*w*/*v*) aqueous preparation was used for Folin–Ciocalteu and ORAC analyses. The liquid aqueous extract showed a total phenolic content, estimated using the Folin–Ciocalteu assay, of 46.6 ± 0.2 mg GAE per 100 mL and an ORAC value of 854.5 ± 1.5 µmol TE per 100 mL (Table 2).

**Figure 1:**
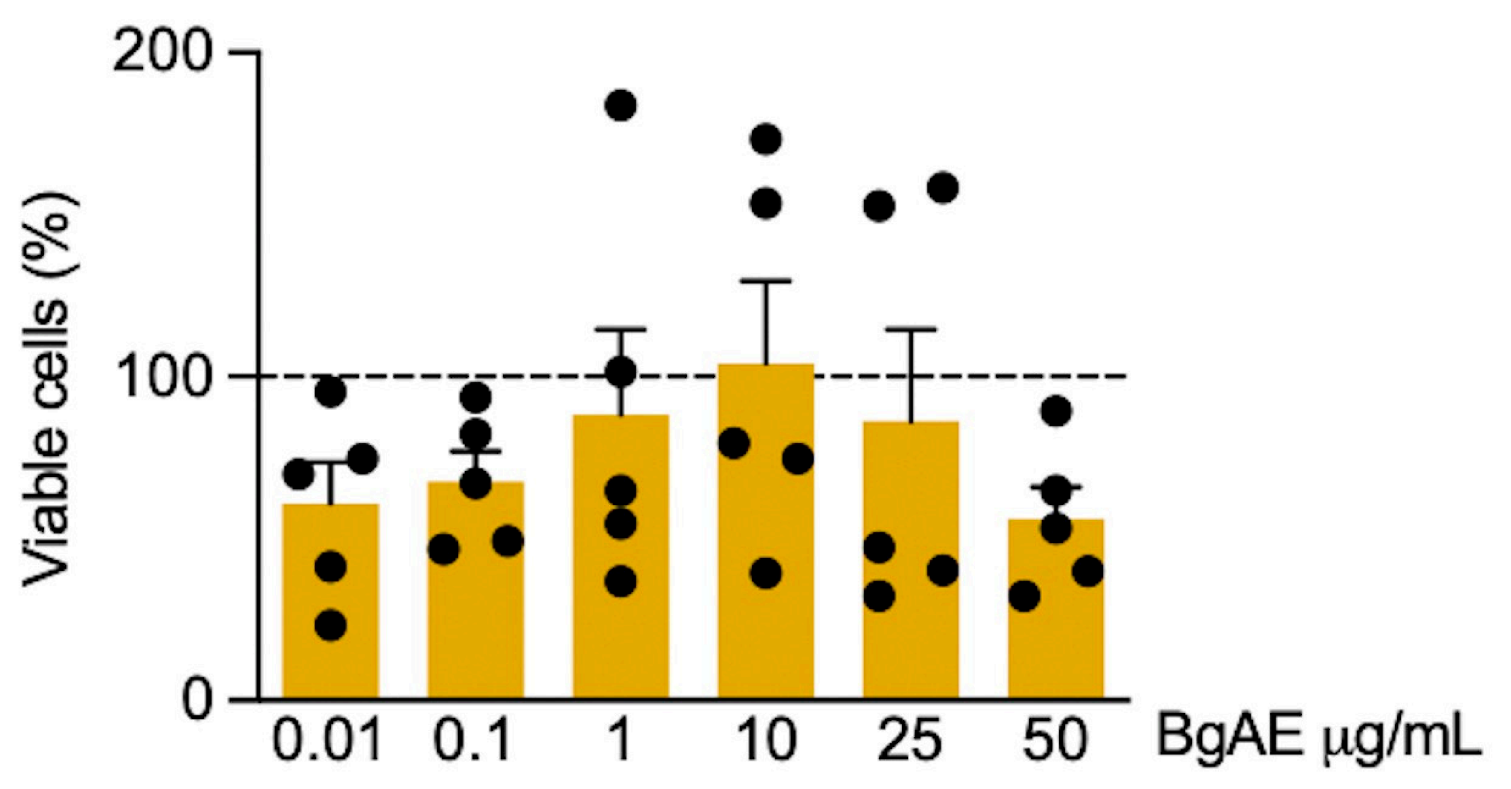
*B. globosa* aqueous extract effect on cell viability in THP-1 cells. Cell viability of THP-1 macrophages measured through trypan blue exclusion after treatment with increasing concentrations of BgAE for 48 h (*n* = 5). Results in the graph are shown as mean ± SEM, with individual data points overlaid. Statistical comparisons were performed using a one-sample *t*-test against the reference value of 100%. Source data are available in Supplementary File S3.

We next assessed the effect of BgAE on the viability of THP-1-derived macrophages. Cells were exposed to BgAE concentrations ranging from 0.01 to 50 µg/mL for 48 h. Under the conditions evaluated, none of the tested concentrations produced a change in cell viability compared with the vehicle-treated control (Figure 1). Based on these results, 0.01, 1, and 50 µg/mL BgAE were selected for subsequent experiments.

### 2.2. BgAE Modulates Hydrogen Peroxide-Induced Senescence-Associated Responses in THP-1-Derived Macrophages

**Figure 2:**
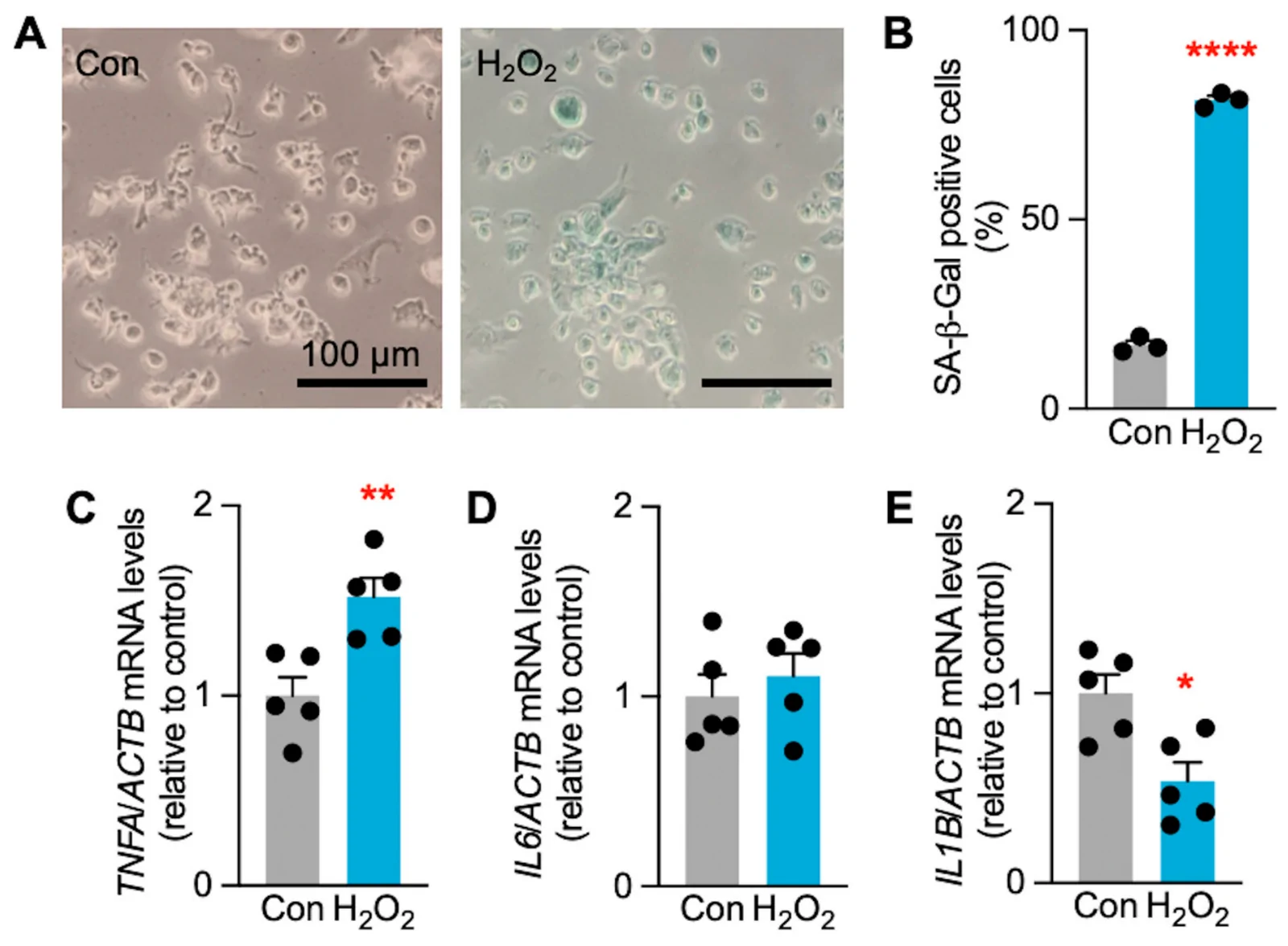
Hydrogen peroxide treatment increases both SA-β-Galactosidase and *TNFA* expression in THP-1 macrophages. (**A**) Representative bright-field images of SA-β-Gal assay in THP-1 cells exposed to vehicle or to 50 µM hydrogen peroxide (H_2_O_2_) for 1 h then allowed to rest for 5 days. (**B**) Quantification of SA-β-Gal-positive cells assessed as in A (*n* = 3). (**C**–**E**) Quantification of the mRNA levels of *TNFA*, *IL6* and *IL1B*, respectively, normalized to *ACTB*, of cells treated as in A (*n* = 5). For each independent imaging experiment, at least 100 cells were analyzed. Scale bars: 100 μm. Results in the graph are shown as mean ± SEM, with individual data points overlaid. Statistical comparisons were performed using an unpaired Student’s *t*-test. * *p* < 0.05, ** *p* < 0.01, and **** *p* < 0.0001, compared with the control condition. All data groups fulfilled the normality and equal variances assumptions. Source data are available in Supplementary File S3.

To induce senescence-associated responses through oxidative stress, THP-1-derived macrophages were exposed to 50 µM H_2_O_2_ for 1 h and subsequently maintained in complete culture medium for 5 days. Compared with vehicle-treated cells, H_2_O_2_ exposure increased the percentage of SA-β-Gal-positive cells and *TNFA* transcript levels, supporting the induction of selected senescence-associated and inflammatory responses (Figure 2A–C). In contrast, IL6 transcript levels were not significantly altered, whereas IL1B expression was decreased following H_2_O_2_ exposure (Figure 2D,E).

**Figure 3:**
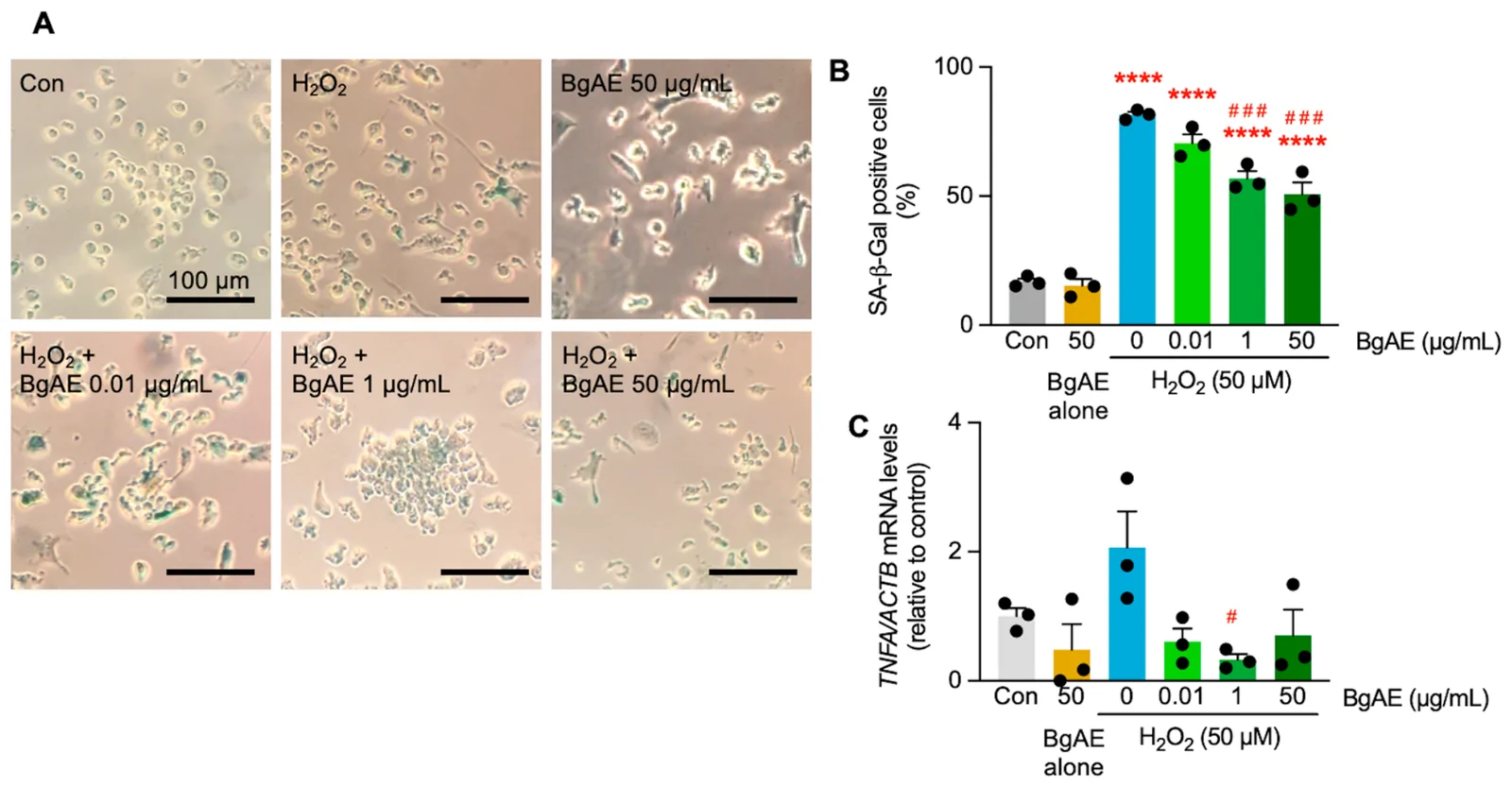
The *Buddleja globosa* aqueous extract modulates H_2_O_2_-induced SA-β-Gal positivity and *TNFA* expression in THP-1-derived macrophages. (**A**) Representative bright-field images of SA-β-Gal staining in vehicle-treated cells, cells treated with 50 µg/mL BgAE alone, and cells exposed to 50 µM H_2_O_2_ with or without pretreatment with 0.01, 1, or 50 µg/mL BgAE. (**B**) Quantification of SA-β-Gal-positive cells under the conditions shown in A (*n* = 3 independent experiments). (**C**) Quantification of TNFA mRNA levels, normalized to ACTB, in vehicle-treated cells, cells treated with 50 µg/mL BgAE alone, and cells exposed to 50 µM H_2_O_2_ with or without pretreatment with 0.01, 1, or 50 µg/mL BgAE (*n* = 3 independent experiments). For each independent imaging experiment, at least 100 cells were analyzed. Scale bars: 100 μm. Results in the graph are shown as mean ± SEM, with individual data points overlaid. Statistical comparisons were performed via ordinary one-way ANOVA. For pairwise comparisons, each experimental group was compared to the control and to the 50 µM H_2_O_2_ condition without BgAE via a Sidak’s post-test. **** *p* < 0.0001 compared with the control condition; # *p* < 0.05 and ### *p* < 0.001 compared with the 50 µM H_2_O_2_-treated condition. All data groups fulfilled the normality and equal variances assumptions. Source data are available in Supplementary File S3.

We next examined whether BgAE modified the senescence-associated responses induced by H_2_O_2_. Pretreatment with 1 and 50 µg/mL BgAE significantly reduced the percentage of SA-β-Gal-positive cells compared with H_2_O_2_ treatment alone, whereas 0.01 µg/mL did not produce a significant reduction (Figure 3A,B). Pretreatment with 1 µg/mL BgAE significantly reduced TNFA transcript levels compared with cells exposed to H_2_O_2_ alone (Figure 3C). A 50 µg/mL BgAE-alone condition was also evaluated for SA-β-Gal positivity and TNFA expression. BgAE alone at this concentration did not significantly alter either SA-β-Gal positivity or TNFA transcript levels relative to vehicle-treated cells (Figure 3B,C).

**Figure 4:**
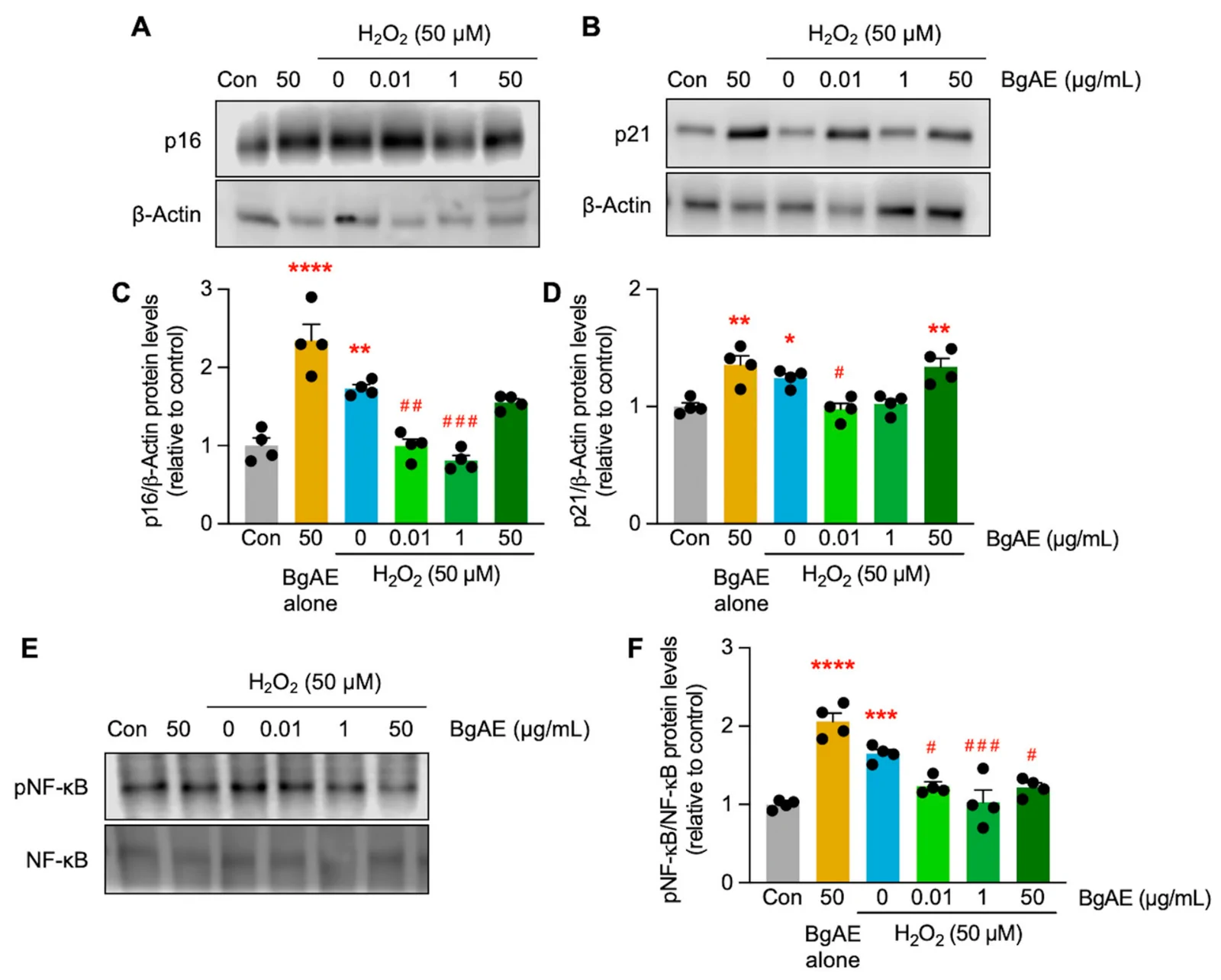
*Buddleja globosa* aqueous extract modulates H_2_O_2_-induced changes in p16, p21, and NF-κB signaling in THP-1-derived macrophages. (**A**,**B**) Representative immunoblots of p16, p21, and β-actin Western blots in THP-1 cells exposed to vehicle or to 50 µM hydrogen peroxide (H_2_O_2_) for 1 h, allowed to rest for 5 days, and then pretreated with increasing amounts of *B. globosa* aqueous extract (BgAE) for 48 h. The 50 µg/mL condition preceding the H_2_O_2_-treated groups corresponds to BgAE administered alone. (**C**,**D**) Quantification of p16 and p21 protein levels, respectively, normalized to β-actin assessed as in A and B (*n* = 4). (**E**) Representative immunoblots of phospho-Ser^536^ p65 NF-κB (pNF-κB), as well as total p65 NF-κB (NF-κB) Western blots, of cells treated as in A. (**F**) Quantification of pNF-κB protein levels, normalized to total NF-κB assessed as in E (*n* = 4). Results in the graph are shown as mean ± SEM, with individual data points overlaid. Statistical comparisons were performed via ordinary one-way ANOVA. For pairwise comparisons, each experimental group was compared to the control and to the 50 µM H_2_O_2_ condition without BgAE via a Sidak’s post-test. * *p* < 0.05, ** *p* < 0.01, *** *p* < 0.001, and **** *p* < 0.0001, compared with the control condition, and # *p* < 0.05, ## *p* < 0.01, ### *p* < 0.001, compared with 50 µM H_2_O_2_ treatment. All data groups fulfilled the normality and equal variances assumptions. Original Western blot images corresponding to the proteins presented in this figure, including experimental-lane identification, indication of the molecular-weight marker, and the location of the bands of interest, are provided in Supplementary File S1. Source data are available in Supplementary File S3.

We then evaluated the effects of BgAE on the senescence-associated proteins p16 and p21 and on NF-κB phosphorylation. Exposure to H_2_O_2_ significantly increased p16 and p21 protein abundance and the phospho-NF-κB/total NF-κB ratio compared with vehicle-treated cells (Figure 4). Pretreatment with 0.01 µg/mL BgAE reduced all three H_2_O_2_-induced responses. The 1 µg/mL concentration reduced p16 abundance and NF-κB phosphorylation but did not significantly modify p21 levels compared with H_2_O_2_ treatment alone. In contrast, pretreatment with 50 µg/mL BgAE reduced NF-κB phosphorylation but did not significantly attenuate the H_2_O_2_-induced changes in p16 or p21. Notably, treatment with 50 µg/mL BgAE in the absence of H_2_O_2_ increased p16 and p21 abundance as well as NF-κB phosphorylation, indicating that the effects of BgAE varied according to concentration and cellular context.

## 3. Discussion

The present study provides initial evidence that an aqueous extract of *Buddleja globosa* modulates several senescence-associated responses induced by oxidative stress in THP-1-derived macrophages. In our experimental model, H_2_O_2_ increased SA-β-Gal positivity, *TNFA* expression, p16 and p21 protein abundance, and NF-κB phosphorylation. Pretreatment with BgAE attenuated a subset of these responses, although the magnitude and direction of the effects varied across the concentrations and markers evaluated. The highest BgAE concentration showed a mixed biological profile. When administered alone, 50 µg/mL BgAE did not significantly alter SA-β-Gal positivity or *TNFA* expression, but it increased p16 and p21 abundance and NF-κB phosphorylation. Thus, the direct effects of the extract at this concentration differed across the endpoints evaluated, further supporting an endpoint- and context-dependent interpretation rather than a uniformly protective or uniformly pro-senescent response. This interpretation is consistent with the current understanding of macrophage senescence as a heterogeneous state that should be evaluated through combinations of complementary markers and whose phenotype varies according to cell type, inducing stimulus, and experimental timing [4,9,22].

The concurrent changes in p16, p21, and NF-κB phosphorylation provide a coordinated pattern across senescence-associated and inflammatory signaling endpoints, but they do not establish a causal mechanistic relationship between these pathways. Because p16 and p21 participate in distinct cell-cycle regulatory programs, their unequal responses across the BgAE concentrations evaluated should not necessarily be interpreted as inconsistent. In a recent in vitro model of macrophage aging, increases in p16 and p21 were accompanied by reduced proliferative activity and increased expression of TNF-α, IL-6, IL-1β, and other SASP-related factors, supporting the interpretation of these proteins as components of a broader senescence-like phenotype rather than interchangeable markers [23]. Similarly, repeated H_2_O_2_ exposure in RAW 264.7 macrophages increased SA-β-Gal activity, p16 and p21 expression, and NF-κB activation, whereas treatment with the polyphenol epigallocatechin gallate attenuated these changes [24]. The reduction in NF-κB phosphorylation observed with BgAE is also consistent with previous macrophage studies, showing that rutin can suppress NF-κB p65 phosphorylation and the production of proinflammatory mediators [16,25]. In the present study, phosphorylation of p65 at Ser536 was used as a signaling-associated readout and should not be interpreted as a direct measure of NF-κB transcriptional activity. NF-κB-dependent transcription was not assessed by nuclear translocation, DNA-binding activity, reporter assays, or pathway inhibition. The absence of a parallel increase in *IL6* despite increased p65 phosphorylation further illustrates that phosphorylation of this site alone does not predict the transcriptional behavior of individual inflammatory targets. Accordingly, the present data support modulation of NF-κB-associated signaling but do not establish NF-κB as the causal mediator of the effects of BgAE. Nevertheless, because BgAE is a complex botanical extract and its constituents were not evaluated individually, the present results do not allow its cellular effects to be attributed to rutin trihydrate, quercetin, or any other single compound [23,24,25]. H_2_O_2_ exposure has been used in macrophage models to induce oxidative stress-associated senescence-like phenotypes. Recent comparative evidence has shown that H_2_O_2_ exposure promotes multiple senescence-associated features in primary macrophages and RAW264.7 cells, including increased SA-β-Gal activity and changes in p16 and p21 signaling [26]. In addition, THP-1-derived macrophages have been reported to exhibit substantial resistance to H_2_O_2_-induced cytotoxicity under oxidative-stress conditions [27]. Together, these observations support the use of H_2_O_2_ as an experimental stressor for investigating senescence-associated responses in macrophages. Nevertheless, the present study evaluated a single recovery time point and did not include a reference senescence inducer or direct assessment of irreversible cell-cycle arrest, proliferative recovery, apoptosis, intracellular ROS, or sustained DNA-damage signaling. Therefore, the current data cannot definitively distinguish stable cellular senescence from a persistent or transient stress-associated phenotype. Future studies incorporating multiple recovery time points and comparison with established senescence-inducing interventions will be required to determine the persistence and reversibility of these responses.

A notable feature of our model was the divergent transcriptional response of the inflammatory genes evaluated. H_2_O_2_ increased *TNFA* expression, whereas IL-6 remained unchanged and IL-1β expression decreased. Therefore, the response observed in THP-1-derived macrophages should not be interpreted as a complete or uniform canonical SASP. The composition of the SASP is dynamic and heterogeneous, varying according to cell type, senescence-inducing stimulus, exposure conditions, and the time at which it is examined [28,29]. Moreover, the establishment of senescence-associated cell-cycle signaling and the development of a secretory phenotype are related but not necessarily synchronous processes [30]. In the present model, cells were exposed to H_2_O_2_ for 1 h and analyzed after a 5-day recovery period, which may have captured a selective or temporally restricted inflammatory response. Because inflammatory mediators were assessed only at the transcript level and at a single time point, our data cannot determine whether IL-6 or IL-1β underwent earlier transient changes or whether their secretion was altered independently of mRNA abundance. Accordingly, these findings support the induction of selected senescence-associated inflammatory responses, rather than a fully developed SASP, and the effects of BgAE should be interpreted as modulation of specific inflammatory markers [28,30].

One of the most informative findings of this study was the non-uniform response observed across the BgAE concentrations evaluated. The 0.01 µg/mL condition produced the most consistent attenuation across the H_2_O_2_-induced protein responses evaluated, whereas 1 µg/mL reduced SA-β-Gal positivity and *TNFA* expression and attenuated selected protein changes. By contrast, 50 µg/mL reduced SA-β-Gal positivity and NF-κB phosphorylation in H_2_O_2_-exposed cells but did not significantly attenuate the H_2_O_2_-induced changes in p16 or p21 and increased p16, p21, and NF-κB phosphorylation when administered alone. Taken together, these findings indicate that the effects of BgAE vary according to concentration, endpoint, and cellular context. Because only three widely separated concentrations were examined across the senescence-associated endpoints, the present study does not define a complete concentration–response relationship or a precise biological activity window.

Recent evidence from a distinct inflammatory model further supports the broader concept that complex botanical preparations can modulate interconnected inflammatory and senescence-associated pathways. Wang et al. reported that a traditional herbal oil modulated BCAA-driven inflammatory senescence in an experimental model of atopic dermatitis [31]. Although the botanical intervention, disease context, and experimental approach differ substantially from those used in the present study, these findings provide complementary contextual evidence that multicomponent botanical preparations can influence inflammatory and senescence-associated signaling.

The phenolic profile and antioxidant capacity of BgAE provide a plausible chemical context for its cellular effects, but they do not establish the underlying mechanism. In particular, ORAC is a cell-free chemical assay and should not be interpreted as evidence of intracellular antioxidant activity or redox regulation. Because extracellular H_2_O_2_ levels were not measured in the presence of BgAE, a contribution from direct chemical scavenging of H_2_O_2_ in the culture medium cannot be excluded, and the observed attenuation of H_2_O_2_-induced responses cannot be attributed exclusively to intracellular redox regulation. Moreover, the present dataset did not include paired chemical and cellular measurements across independently varying extract preparations and therefore does not support a meaningful correlation analysis between individual phenolic constituents and cellular endpoints. Importantly, the six compounds quantified by HPLC-DAD represent only a targeted subset of the phytochemical constituents potentially present in BgAE. Other secondary metabolites previously described in *B. globosa*, including phenylethanoid glycosides and additional flavonoid and phenolic derivatives, were not comprehensively characterized in the present study and may also contribute to the observed biological responses. Among the compounds quantified by HPLC-DAD, the BgAE profile was characterized primarily by flavonol-related compounds, particularly rutin trihydrate and quercetin, together with caffeic acid and kaempferol-3-rutinoside. This profile was broadly consistent with previous phytochemical characterizations of *B. globosa* leaf material. Torres-Vega et al. identified caffeoyl derivatives, verbascoside-related phenylpropanoids, quercetin glycosides, and other flavonoid constituents in *B. globosa* leaves [22]. Although the extraction procedures and chromatographic conditions differed between studies, including the column, gradient, and detection system, the occurrence of related phenolic compound classes provides complementary chemical consistency with the botanical material identified as *B. globosa*. Direct retention-time comparison between studies was not performed; therefore, this comparison should be interpreted at the level of phenolic compound classes rather than as compound-level or taxonomic confirmation. Consistent with this phytochemical profile, previous work showed that an aqueous leaf extract of *B. globosa* protected H_2_O_2_-challenged fibroblasts, with flavonoids and caffeic acid derivatives proposed to contribute to its antioxidant activity [32]. However, direct comparisons with the present study should be made cautiously because of differences in the plant material, extract preparation, cellular model, and experimental design. Recent evidence from other medicinal botanical preparations further illustrates the value of combining comprehensive chemical profiling with functional biological evaluation. Shi et al. used UHPLC-Q-Exactive Orbitrap mass spectrometry to achieve extensive chemical characterization of *K. coccinea* leaf extracts and complemented this analysis with antioxidant and anti-inflammatory assays [33]. Although the botanical species and experimental models differ from those used in the present study, this integrated approach highlights the importance of coupling detailed phytochemical characterization with biological assessment when investigating complex plant extracts.

Several strengths and limitations should be considered when interpreting these findings. Additional limitations concern the botanical and chemical standardization of the preparation. Leaves and stems were pooled without a predefined anatomical-part ratio, the exact collection date and phenological stage were not formally recorded, and no herbarium voucher specimen was deposited. Because leaves and stems were not extracted, chemically characterized, or biologically evaluated separately, their individual contributions to the observed cellular effects cannot be determined from the present study. Although botanical identification was supported by field recognition, expert input, taxonomic information available for the Chilean flora, and broad chemical consistency with previously reported phytochemical profiles of *B. globosa*, these complementary lines of evidence do not substitute for formal taxonomic authentication. In addition, extraction yield and dry-solids content were not determined through a formal batch-level standardization procedure, and stability testing was not performed. These limitations should be considered when assessing the reproducibility and chemical standardization of BgAE and preclude considering the preparation as a fully standardized botanical extract. The use of a water-based preparation provides ethnomedicinal relevance because aqueous infusions represent a traditional form of *B. globosa* use. However, the preparation evaluated in the present study should not be considered an exact or standardized reproduction of a traditional formulation, and its biological effects should therefore be interpreted within the context of the experimental preparation used here. A strength of the study was the evaluation of multiple senescence-associated and inflammatory endpoints across different extract concentrations. Nevertheless, THP-1-derived macrophages represent a simplified and reproducible in vitro model and do not fully reproduce the phenotypic and functional diversity of primary human macrophages [34]. Our conclusions are also limited using a single oxidative stressor, one post-exposure time point, and a selected panel of senescence-associated markers. Although SA-β-Gal, p16, and p21 were evaluated together, the study did not directly assess post-treatment viability or cell number after the 5-day recovery period, irreversible cell-cycle arrest or proliferative capacity, apoptosis, sustained DNA-damage signaling, intracellular reactive oxygen species, mitochondrial function, or changes in macrophage activity. Moreover, inflammatory mediators were measured at the transcript level rather than as secreted proteins. Similarly, NF-κB pathway activity was inferred from p65 Ser536 phosphorylation without direct assessment of nuclear translocation, DNA binding, transcriptional reporter activity, or pharmacological pathway inhibition. Therefore, the present study cannot establish that NF-κB signaling is mechanistically required for the cellular effects of BgAE. The absence of a reference intervention limits direct comparison of the magnitude of the BgAE effects against established antioxidant or senescence-modulating agents. However, the present study was designed to evaluate the integrated biological activity of the aqueous botanical preparation rather than to establish efficacy relative to an isolated antioxidant or senomorphic agent. This distinction is relevant because the biological activity of natural-product extracts may arise from interactions among multiple constituents, including additive, synergistic, or antagonistic effects [35]. Accordingly, comparison with a single purified compound would not necessarily represent a mechanistically equivalent comparator. Overall, the present findings support modulation of selected senescence-associated responses but do not establish irreversible cellular senescence. These complementary endpoints should be prioritized in future studies to more comprehensively distinguish stable senescence from persistent or transient stress-associated responses. These limitations prevent us from defining BgAE as a senolytic or senomorphic intervention and from extrapolating the findings to primary macrophages, animals, or humans. Nevertheless, the coordinated changes observed across complementary endpoints support BgAE as a relevant botanical preparation for further investigation in oxidative stress-associated macrophage senescence.

Taken together, our findings extend the reported antioxidant and anti-inflammatory properties of *B. globosa* by showing that an aqueous preparation can also modulate selected senescence-associated responses in oxidatively stressed THP-1-derived macrophages. The relevance of the study lies not in assigning the observed effects to a single phenolic compound, but in evaluating the integrated biological activity of a water-based botanical preparation within the broader context of the traditional aqueous use of matico. The different responses observed across the concentrations evaluated emphasize that natural extracts should not be assumed to be uniformly beneficial and highlight the need for denser concentration-response studies to more precisely define their biological activity window. In this context, BgAE should be regarded as a candidate botanical preparation for further investigation rather than as an established senotherapeutic intervention. Future studies should prioritize improved botanical and chemical standardization of BgAE, including formal taxonomic authentication and deposition of voucher specimens, documentation of collection date and phenological stage, predefined plant-part proportions and separate chemical and biological evaluation of the individual plant tissues, validated stability assessment, and batch-to-batch chemical characterization, together with more comprehensive high-resolution mass spectrometry-based phytochemical profiling to identify additional constituents potentially contributing to the biological activity of BgAE. Future mechanistic studies should also directly assess intracellular redox regulation, including ROS generation, glutathione redox status, oxidative-damage markers, mitochondrial function, and redox-responsive signaling pathways such as Nrf2. Experimental designs incorporating removal of BgAE before H_2_O_2_ exposure and direct quantification of extracellular H_2_O_2_ should help distinguish extracellular chemical scavenging from cell-mediated redox responses. Further work should also incorporate complementary markers of persistent DNA damage, including γH2AX and 53BP1, direct functional assessment of irreversible cell-cycle arrest and proliferative capacity, and quantification of secreted SASP-associated cytokines and chemokines. Mechanistic studies should additionally determine whether the observed changes depend on NF-κB signaling using complementary approaches such as pathway inhibition, assessment of p65 nuclear translocation or DNA-binding activity, and NF-κB-responsive reporter assays. These analyses will be important to distinguish stable cellular senescence from persistent stress-associated responses and to characterize the associated inflammatory phenotype more comprehensively. Validation of the main findings in primary human monocyte-derived macrophages will also be important to determine their reproducibility in a biologically more representative cellular model. Such approaches will be necessary to determine whether the effects observed here are reproducible across biological models and to clarify the mechanisms through which *B. globosa* influences macrophage responses to oxidative stress.

Individual phenolic compound concentrations were not directly measured in the cellular treatment medium. Nevertheless, using the Folin–Ciocalteu-derived total phenolic content and the estimated dry-solids concentration of the same aqueous preparation, we retrospectively estimated the total phenolic-equivalent exposure associated with the BgAE concentrations used in the cellular experiments (Supplementary Table S2). These estimates should be interpreted cautiously because they are expressed as gallic acid equivalents and do not represent measured concentrations of individual BgAE constituents. Accordingly, they provide contextual information on the experimental exposure but do not establish whether specific bioactive compounds reach physiologically or pharmacologically relevant concentrations in vivo.

Moreover, the present study did not assess the stability, absorption, metabolism, or tissue distribution of the individual phenolic constituents identified in BgAE. Therefore, the parent compounds detected by HPLC-DAD may not necessarily correspond to the chemical species or concentrations reaching target tissues after administration. Dedicated pharmacokinetic studies evaluating systemic and tissue exposure to parent compounds and relevant metabolites will be required before the in vitro concentrations used here can be interpreted in a translational or therapeutic context.

Because BgAE was administered before H_2_O_2_ exposure, the present design represents a pretreatment paradigm and evaluates modulation of the cellular response to a subsequent oxidative challenge rather than reversal or regulation of an already established senescent phenotype. Accordingly, the present findings do not establish senomorphic or senotherapeutic activity. Demonstrating such activity would require post-induction treatment after a stable senescence-associated state had first been established and functionally characterized.

**Figure 5:**
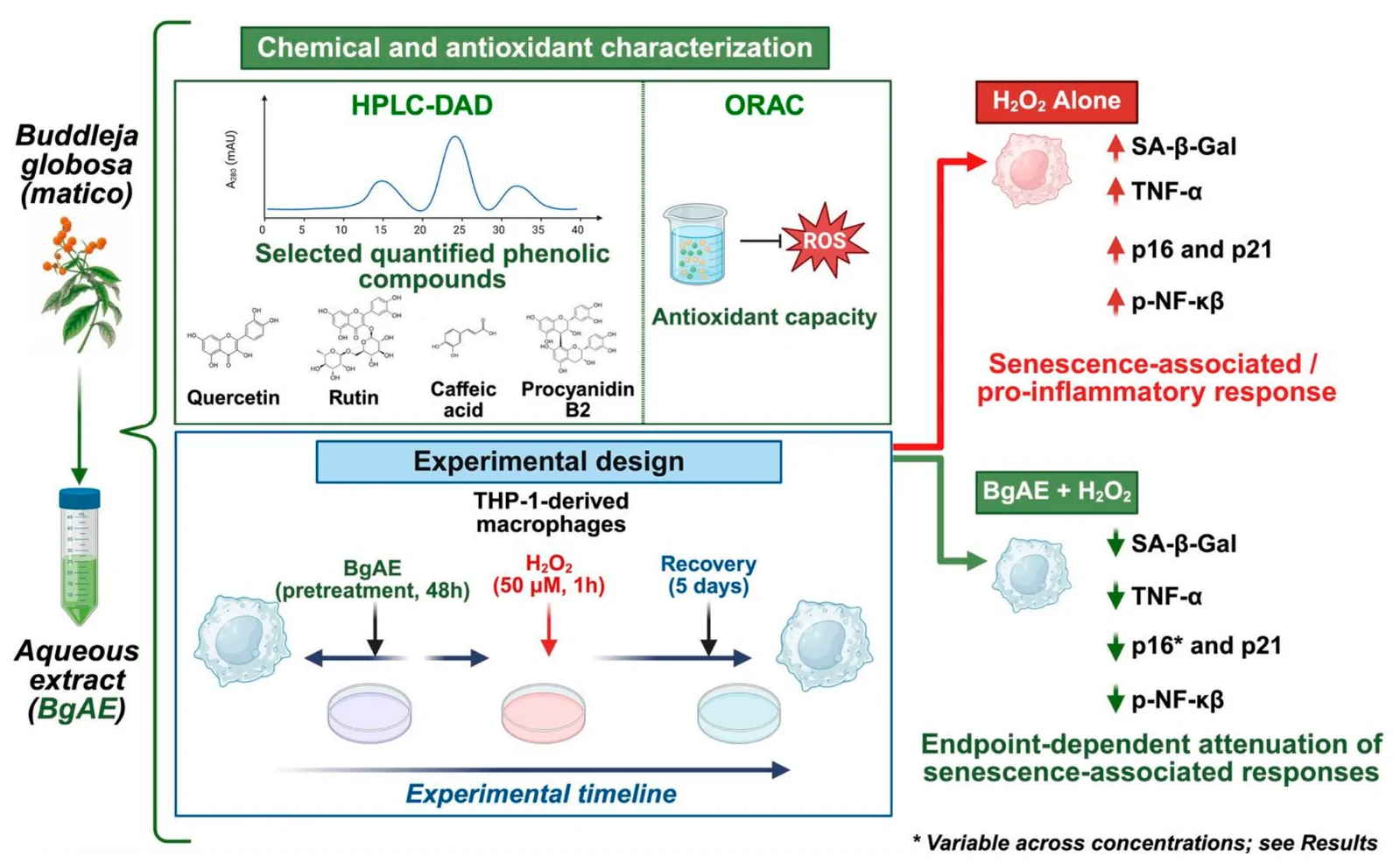
Summary of the chemical characterization, experimental design, and concentration-, endpoint-, and context-dependent effects of BgAE on selected oxidative stress-induced senescence-associated responses in THP-1-derived macrophages.

In conclusion, the aqueous extract of *Buddleja globosa* modulated the specific molecular and phenotypic endpoints evaluated in H_2_O_2_-exposed THP-1-derived macrophages, including SA-β-Gal positivity, TNFA expression, p16 and p21 abundance, and NF-κB phosphorylation. The magnitude and consistency of these effects varied according to concentration and endpoint. As summarized in Figure 5, BgAE did not exert a uniformly protective effect; rather, its effects varied according to the concentration, endpoint, and cellular context evaluated. The 0.01 and 1 µg/mL conditions generally showed the most consistent attenuation of H_2_O_2_-induced changes, whereas the highest concentration displayed a mixed profile and increased p16, p21, and NF-κB phosphorylation when administered in the absence of oxidative stress. Overall, these findings provide initial in vitro evidence that a water-based preparation of *B. globosa* can modulate selected molecular and phenotypic responses associated with H_2_O_2_ exposure in THP-1-derived macrophages and support further investigation of this aqueous botanical preparation.

## 4. Materials and Methods

### 4.1. Buddleja Globosa Aqueous Extract Preparation

Samples of *Buddleja globosa*, consisting of leaves and stems, were collected during spring from Centro Ceremonial Parque Mahuidache, located in Comuna de El Bosque, Santiago Metropolitan Region, Chile (33.568° S, 70.681° W). Leaves and stems were pooled without a predefined mass ratio. The exact collection date and phenological stage of the collected plants were not formally recorded. Plant selection and field identification as matico (*B. globosa*) were performed with the assistance of a local herbalist familiar with the medicinal flora of the area (Manuel Manríquez), a social anthropologist familiar with the local ethnobotanical context (José Pinto) and a researcher with expertise in plant genomics (Igor Pacheco, Ph.D.). The taxonomic assignment was additionally cross-checked against the Catalogue of the Vascular Plants of Chile, which recognizes *B. globosa* Hope as a native shrub occurring in the Metropolitan Region of Santiago and lists “matico” among its common names [36]. No herbarium voucher specimen was deposited, and no molecular taxonomic identification was performed. The samples were dried at 60 °C for 16 h in a conventional oven and homogenized using an A11 basic S1 grinder (IKA, Staufen, Germany) at 37,000 × g for 10 s. Aqueous extracts were prepared using 10 g of dried ground material in 200 mL of distilled water (5% *w*/*v*) previously heated to 100 °C and allowed to steep for 60 min, after which the infusions were centrifuged at 8000 *g* for 15 min at 4 °C. Finally, samples were filtered through a 0.2 μm membrane and stored at –80 °C until use. The filtered aqueous extract was frozen at −80 °C and subsequently lyophilized to dryness. The resulting lyophilized material was maintained at −80 °C until use as a precaution to minimize degradation during storage. No formal stability study of the preparation was performed. Aliquots of this aqueous preparation were used for HPLC-DAD, Folin–Ciocalteu, and ORAC analyses. For the cellular experiments, an aliquot of the same aqueous preparation was lyophilized to dryness. Based on the lyophilization of a 3 mL aliquot, which yielded approximately 52 mg of dried extract solids, the dry-solids concentration of the aqueous preparation was estimated to be approximately 17.3 mg/mL. This corresponds to an apparent extraction yield of approximately 34.7% (*w*/*w*) relative to the dried botanical material used for extraction. Because this estimate was derived from an aliquot rather than from a formal batch-yield determination, it should be considered approximate. For cellular experiments, the lyophilized material was reconstituted at 50 mg/mL in sterile fetal bovine serum-free RPMI culture medium. All BgAE concentrations reported in the cellular experiments (0.01–50 μg/mL) refer to the mass of lyophilized aqueous-extract solids per milliliter of the final culture medium.

### 4.2. HPLC-DAD Analysis of Phenolic Compounds

Phenolic compounds present in the BgAE were identified and quantified by high-performance liquid chromatography (HPLC) using a Hitachi Chromaster 5000 series system (Tokyo, Japan) equipped with a photodiode array detector and the Chromaster System Manager V1.2 software for data acquisition. Chromatographic separation was performed on a reversed-phase RP-18 Purospher STAR column (250 × 4.6 mm i.d.) maintained at 35 °C, with a 10 µL injection volume. Two gradient elution programs were applied using three mobile phases: methanol (A), acetonitrile (B), and 0.1% formic acid in water (C). The first gradient program, used for the separation of chlorogenic, syringic, caffeic, ferulic, sinapic, abscisic, trans-cinnamic, and coumaric acids, as well as catechin, rutin trihydrate, daidzein, quercetin, naringenin, genistein, apigenin, kaempferol, isorhamnetin, pinocembrin, chrysin, and galangin, was as follows: 0–10 min 20% B/80% C; 10.1–40 min 7.5% A/25% B/67.5% C; 40.1–50 min 15% A/25% B/60% C; 50.1–65 min 15% A/45% B/40% C; 65.1–80 min 20% B/80% C, at a flow rate of 0.8 mL/min. A second gradient program was applied for the resolution of gallic, protocatechuic, and vanillic acids, epicatechin, procyanidins B1 and B2, kaempferol-3-rutinoside, isoquercitrin, taxifolin, epigallocatechin, and epicatechin gallates: 0–30 min 15% A/85% B; 30.1–45 min 25% B/75% C; 45.1–55 min 40% B/60% C; 55.1–60 min 50% B/50% C; 60.1–65 min 80% B/20% C; 65.1–75 min 15% B/85% C, at a flow rate of 1.0 mL/min. Spectrophotometric detection was carried out using a diode-array detector scanning from 210 to 700 nm. Chromatograms used for compound quantification were monitored at 370 nm for caffeic acid, rutin trihydrate, and quercetin, and at 290 nm for procyanidin B2, kaempferol-3-rutinoside, and epicatechin gallate. Compound identification was based on retention-time matching and UV–visible spectral comparison with reference standards. For quantification, individual calibration curves were constructed using a multistandard mixture containing equal concentrations of each compound across a range of 2–250 µM. All calibration curves showed R^2^ values >0.99. The functional limit of quantification (LOQ) was 2 µM, corresponding to the lowest concentration included in the calibration range. Compound-specific limits of detection were not re-determined in the present analysis; previously reported detection limits for this analytical approach ranged from 2 to 133 µg/g, depending on the compound [37]. Representative HPLC-DAD chromatograms showing the separation and retention times of the quantified compounds are provided in Supplementary Figure S1, and the corresponding analytical information is summarized in Supplementary Table S1. HPLC-DAD measurements were performed in triplicate. The sample analyzed corresponded to the original 5% (*w*/*v*) aqueous preparation and was analyzed in liquid form without prior lyophilization. Quantitative results were expressed as mg per 100 g of dried botanical material used to prepare the aqueous extract.

### 4.3. Total Phenolic Content and Oxygen Radical Absorbance Capacity

Total phenolic content in BgAE was estimated using the Folin–Ciocalteu (F–C) assay. Briefly, 15 μL of each sample, diluted in water, or standard was mixed with 200 μL of a solution containing the F–C reagent, previously diluted 1:10 (*v*/*v*) in distilled water, 40 μL of sodium carbonate (20% *w*/*v*), and 45 μL of distilled water. After incubation at 37 °C for 30 min, absorbance at 765 nm was measured in a 96-well plate using a multimode microplate reader. The ORAC assay was performed according to the internal procedure of the Laboratory of Antioxidants (MME-Pro-002), which is based on a previously described method [38]. For this, AAPH was used as a source of peroxyl radicals and fluorescein as an oxidizable probe. A 45 μL aliquot of BgAE, diluted in 7.5 × 10^−2^ M sodium phosphate buffer at pH 7.4, was added to 96-well microplates containing AAPH (1.8 × 10^−2^ M) and fluorescein (1.08 × 10^−7^ M). The plates were placed in a Multi-Mode Microplate Reader (Synergy HT) and incubated for 60 min at 37 °C with shaking of the plates every 3 min. Because the Folin–Ciocalteu reagent can also react with non-phenolic reducing compounds, the resulting values were interpreted as an estimate of total phenolic content expressed as gallic acid equivalents (GAEs), rather than as a compound-specific measurement of phenolics. Potential contributions from non-phenolic reducing constituents were not independently quantified in the present preparation. The results of ORAC activity were estimated based on a standard curve of Trolox, using a quadratic regression equation obtained between the Trolox concentration and net area under the fluorescence decay curve. Total phenolic content and ORAC measurements were performed in duplicate in three independent analytical determinations.

### 4.4. THP-1 Cell Culture

The human monocyte cell line THP-1 (TIB-202, ATCC, Manassas, VA, USA) was cultured in RPMI 1640 medium (Sigma-Aldrich, St. Louis, MO, USA) supplemented with 10% fetal bovine serum (Biological Industries, Kibbutz Beit-Haemek, Israel) and antibiotics (penicillin–streptomycin) at 37 °C in a controlled-atmosphere incubator with 5% CO_2_. For experiments, cells were seeded at a density of 100,000 cells/cm^2^. The medium was replaced every 2–3 days.

To induce differentiation, THP-1 monocytes were cultured in RPMI medium containing 100 nM phorbol 12-myristate 13-acetate (Sigma-Aldrich) for 24 h, as previously published [39]. The medium was then removed and replaced with complete RPMI medium 24 h prior to initiating the experiments. Cell morphology and adhesion, used as morphological indicators of macrophage differentiation status, were monitored by visual inspection using bright-field microscopy.

Differentiated THP-1 macrophages were either pretreated or not with BgAE for 48 h and subsequently exposed to 50 μM H_2_O_2_ for 1 h to induce senescence-associated responses through oxidative stress. After exposure, cells were washed twice with PBS and subsequently incubated for 5 days in a complete RPMI medium. The medium was replaced every 2 days. Cell morphology and adhesion, used as morphological indicators of macrophage differentiation status, were monitored during the 5-day recovery period by visual inspection using bright-field microscopy. Culture sample sets obtained on different days were considered independent experiments (biological replicates), whereas culture samples obtained on the same day and treated in parallel were considered technical replicates. All independent cellular experiments were conducted using the same BgAE preparation rather than independently prepared extract batches; therefore, the present design does not assess batch-to-batch extract variability.

### 4.5. Cell Viability Assay

To determine the appropriate concentration of BgAE to use, preliminary experiments were performed. Differentiated macrophages were treated with 0.01, 0.1, 1, 10, 25, or 50 μg/mL of BgAE for 48 h. After incubation, cells were trypsinized and cell viability was assessed by 0.4% trypan blue exclusion using a TC20 automated cell counter (Bio-Rad, Hercules, CA, USA). Assays were performed in duplicate.

### 4.6. SA-β-Galactosidase Assay

Cells were treated with a senescence-associated β-galactosidase staining kit (Cell Signaling Technology, Danvers, MA, USA) according to the manufacturer’s instructions. After overnight incubation at 37 °C, cells were examined under a microscope, and the percentage of SA-β-gal-positive cells was quantified. Three different fields containing 100 or more cells were photographed, and the number of X-gal-stained blue cells at pH 6.0 was divided by the total number of cells per field.

### 4.7. Reverse Transcription Quantitative PCR (RT-qPCR)

Cells were lysed using TRIzol reagent (Invitrogen, Carlsbad, CA, USA) for RNA extraction with the PureLink Genomic RNA Kit (Invitrogen, Carlsbad, CA, USA), following the manufacturer’s instructions. Reverse transcription was performed using the High-Capacity cDNA Reverse Transcription Kit (Applied Biosystems, Foster City, CA, USA). mRNA expression was evaluated with the StepOne Real-Time PCR System (Applied Biosystems) using the SYBR FAST qPCR Kit (Applied Biosystems, Foster City, CA, USA). The thermal cycling protocol consisted of a pre-incubation step at 95 °C for 20 s, followed by 40 cycles at 95 °C for 3 s and 60 °C for 30 s. The following specific primer sequences were used: *TNFA*: CCA GGC AGT CAG ATC ATC TTC TC (forward) and AGC TGG TTA TCT CTC AGC TCC AC (reverse); *IL6*: CAA TCT GGA TTC AAT GAG GAG AC (forward) and CTC TGG CTT GTT CCT CAC TAC TC (reverse); *IL1B*: GGA CAA GCT GAG GAA GAT GC (forward) and TCG TTA TCC CAT GTG TCG AA (reverse); *ACTB*: AGA GCC TCG CCT TTG CCG ATC C (forward) and GAC GAC GAG CGC GGC GAT ATC (reverse). Primer efficiencies were 106.8% for *TNFA*, 97.2% for *IL6*, 91.5% for *IL1B*, and 90.2% for *ACTB*. Reactions were performed in duplicate, including a negative control lacking cDNA. These data were then averaged to obtain one value per independent experiment. Results were normalized to *ACTB*. Relative mRNA expression was determined using the Pfaffl method [40]. Original qPCR files corresponding to the mRNAs presented in Figure 2 and Figure 3 are provided in Supplementary File S2.

### 4.8. Western Blot

Cell lysis was performed to obtain total proteins. NP-40 lysis buffer (10 mM Tris, 150 mM NaCl, 0.01% Nonidet P-40, pH 8.0, 10% glycerol) supplemented with protease (Roche 04 693 124 001, Sigma-Aldrich, St. Louis, MO, USA) and phosphatase inhibitors (Roche 04 906 845 001, Sigma-Aldrich, St. Louis, MO, USA) were used. Following scraping, lysates were collected into microcentrifuge tubes and centrifuged at 12,000 *g* for 15 min at 4 °C. Supernatants were recovered, and protein concentration was determined using a BCA Protein Assay Kit (Pierce 23225, Thermo Fisher Scientific, Waltham, MA, USA), according to the manufacturer’s instructions. Approximately 40 µg of total protein lysate were subjected to SDS–polyacrylamide gel electrophoresis with 12% resolving gel and 4% stacking gel. Proteins were electrotransferred to a 0.2 μm nitrocellulose membrane using a transfer buffer (24 mM Tris, 194 mM glycine, 20% methanol). Membranes were blocked with a TBS buffer (20 mM Tris, 150 mM NaCl, pH 7.4) containing 5% BSA and 0.1% Tween for 1 h at room temperature, and subsequently incubated overnight (16 h) at 4 °C with one of the following primary antibodies: anti-p16 (#18769, Cell Signaling Technology, Danvers, MA, USA; 1:1000), anti-p21 (#37543S, Cell Signaling Technology, Danvers, MA, USA; 1:1000), anti-phospho-Ser^536^ p65 NF-κB (#3033S, Cell Signaling Technology, Danvers, MA, USA; 1:1000), anti-p65 NF-κB (#8242S, Cell Signaling Technology, Danvers, MA, USA; 1:1000), or anti-β-actin (sc81178, Santa Cruz Biotechnology, Dallas, TX, USA; 1:5000). Equal aliquots of the same protein lysates from all experimental conditions included in each comparison were resolved under the same electrophoretic conditions. p16 and p21 were analyzed on separate membranes, with β-actin detected on the corresponding membrane and used for normalization of each target protein. For NF-κB analysis, phospho-Ser536 p65 NF-κB was first detected, after which the same membrane was stripped and reprobed for total p65 NF-κB. Phospho-Ser536 p65 NF-κB levels were normalized to total p65 NF-κB. Target protein levels were normalized to β-actin analyzed in the corresponding membrane, whereas phospho-Ser536 p65 NF-κB levels were normalized to total p65 NF-κB. After three washes for 10 min each with TBS-Tween, membranes were incubated for 1 h at room temperature with horseradish peroxidase-conjugated secondary antibodies against rabbit or mouse IgG (#49590 or #47606, Rockland Immunochemicals, Limerick, PA, USA), according to the host species of each primary antibody. Detection of immune complexes was performed by chemiluminescence using the Pierce ECL Substrate Kit (Thermo Fisher Scientific, Waltham, MA, USA). The luminescent signal was digitized with a LI-COR C-Digit 3600 scanner (LI-COR Biosciences, Lincoln, NE, USA), and band intensity was quantified using ImageJ software version 1.51j8 (National Institutes of Health, MD, USA).

Original Western blot images corresponding to the proteins presented in Figure 4 are provided in Supplementary File S1. Experimental conditions are identified in the corresponding lanes, and the molecular-weight marker and expected position of each protein of interest are indicated to facilitate correspondence with the representative immunoblots shown in Figure 4. Any brightness or contrast adjustment used for figure preparation was applied uniformly to the entire image, without selective modification of individual lanes or bands.

### 4.9. Statistical Analysis

Results are shown as mean ± SEM, with individual data points overlaid. Unless otherwise specified, *n* denotes the number of independent biological experiments rather than technical replicate measurements. Technical replicates were not treated as independent observations, and their assay-specific handling is described in the corresponding methodological sections. No prospective sample-size calculation or formal power analysis was performed; the reported *n* values correspond to the number of independent biological experiments completed for each endpoint in the original experimental dataset. Comparisons between two independent groups were performed using an unpaired Student’s *t*-test. For the cell-viability analysis, normalized values were compared with the reference value of 100% using a one-sample *t*-test. For RT-qPCR experiments, statistical comparisons were performed using the individual relative-expression (fold-change) values calculated for each independent experiment. For comparisons involving more than two groups, ordinary one-way ANOVA followed by Sidak’s multiple-comparisons test was used. Each experimental group was compared with the control and with the 50 µM H_2_O_2_ condition without BgAE, as indicated in the corresponding figure legends. A two-way factorial analysis was not applied because the experimental design did not include all combinations of H_2_O_2_ exposure and BgAE concentration; specifically, BgAE-alone conditions were not systematically evaluated at each concentration. Accordingly, the observed treatment conditions were analyzed as a single factor, and no formal H_2_O_2_ × BgAE concentration interaction was inferred. Prior to parametric analyses, normality was assessed using the Shapiro–Wilk test. Homogeneity of variance was evaluated using the F test for two-group comparisons and the Brown–Forsythe test for analyses involving more than two groups.

## Figures and Tables

**Table 1:** Phenolic compounds quantified by HPLC-DAD in the *Buddleja globosa* aqueous extract (BgAE) prepared at 5% (*w*/*v*). Values are expressed as mg per 100 g of dried botanical material used to prepare the aqueous extract and are presented as mean ± SD from three analytical determinations (*n* = 3).

Compound	Quantity (mg/100 g Dried Botanical Material)
Rutin trihydrate	489.3 ± 8.5
Quercetin	171.0 ± 1.9
Caffeic acid	107.3 ± 14.1
Procyanidin B2	75.2 ± 10.92
Kaempferol-3-rutinoside	23.1 ± 1.2
Epicatechin gallate	3.2 ± 0.2

**Table 2:** Total phenolic content and antioxidant capacity of the 5% (*w*/*v*) *Buddleja globosa aqueous* extract (BgAE). Values were determined in the liquid aqueous preparation and are expressed per 100 mL of extract (*n* = 3).

Sample	Total Phenolic Content (mg GAE/100 mL Aqueous Extract)	ORAC (µmol TE/100 mL Aqueous Extract)
BgAE	46.6 ± 0.2	854.5 ± 1.5

## Data Availability

The original data presented in the study are openly available in Zenodo at DOI: [https://doi.org/10.5281/zenodo.22711842]. The original contributions presented in this study are included in the article/Supplementary Material. Further inquiries can be directed to the corresponding author.

## Supplementary Materials

The following supporting information can be downloaded at: https://doi.org/10.5281/zenodo.22711842. Supplementary Figure S1: representative HPLC-DAD chromatograms; Supplementary Table S1: HPLC-DAD analytical information; Supplementary Table S2: estimated total phenolic-equivalent exposure in cellular experiments; Supplementary File S1: Original Western Blot Images; Supplementary File S2: Original qPCR Data Files; Supplementary File S3: Source Data.

## Abbreviations

The following abbreviations are used in this manuscript:

*ACTB*	beta-actin
ATCC	American Type Culture Collection
BCA	bicinchoninic acid
BgAE	*Buddleja globosa* aqueous extract
BSA	bovine serum albumin
cDNA	complementary DNA
CO_2_	carbon dioxide
ECL	enhanced chemiluminescence
FBS	fetal bovine serum
GAE	gallic acid equivalent
GSH	reduced glutathione
GSSG	oxidized glutathione
H_2_O_2_	hydrogen peroxide
HPLC-DAD	high-performance liquid chromatography with diode-array detection
IL-1β	interleukin-1 beta
IL-6	interleukin-6
NF-κB	nuclear factor kappa B
NP-40	Nonidet P-40
ORAC	oxygen radical absorbance capacity
PBMCs	peripheral blood mononuclear cells
p16	cyclin-dependent kinase inhibitor 2A (*p16*^INK4a^)
p21	cyclin-dependent kinase inhibitor 1A (*p21*^Cip1/Waf1^)
pNF-κB	phosphorylated NF-κB (Ser536)
qPCR	quantitative polymerase chain reaction (real-time PCR)
RPMI 1640	Roswell Park Memorial Institute 1640 medium
ROS	reactive oxygen species
RT-PCR	reverse transcription polymerase chain reaction
SA-β-Gal	senescence-associated β-galactosidase
SASP	senescence-associated secretory phenotype
SDS	sodium dodecyl sulfate
TBS	Tris-buffered saline
TE	Trolox equivalents
TNF-α	tumor necrosis factor alpha
X-gal	5-bromo-4-chloro-3-indolyl β-D-galactopyranoside
